# Opposing effects of Notch-signaling in maintaining the proliferative state of follicle cells in the telotrophic ovary of the beetle *Tribolium*

**DOI:** 10.1186/1742-9994-9-15

**Published:** 2012-08-06

**Authors:** Daniel Bäumer, Nadi M Ströhlein, Michael Schoppmeier

**Affiliations:** 1 Department Biology, Developmental Biology Unit, Erlangen, University, Staudstr. 5, Erlangen, 91058, Germany

**Keywords:** Tribolium, Telotrophic oogenesis, Follicle cells, Axis formation, Notch-signaling

## Abstract

**Introduction:**

Establishment of distinct follicle cell fates at the early stages of *Drosophila* oogenesis is crucial for achieving proper morphology of individual egg chambers. In *Drosophila* oogenesis, Notch-signaling controls proliferation and differentiation of follicular cells, which eventually results in the polarization of the anterior-posterior axis of the oocyte. Here we analyzed the functions of *Tribolium* Notch-signaling factors during telotrophic oogenesis, which differs fundamentally from the polytrophic ovary of *Drosophila*.

**Results:**

We found Notch-signaling to be required for maintaining the mitotic cycle of somatic follicle cells. Upon *Delta* RNAi, follicle cells enter endocycle prematurely, which affects egg-chamber formation and patterning. Interestingly, our results indicate that *Delta* RNAi phenotypes are not solely due to the premature termination of cell proliferation. Therefore, we monitored the terminal/stalk cell precursor lineage by molecular markers. We observed that upon *Delta* RNAi terminal and stalk cell populations were absent, suggesting that Notch-signaling is also required for the specification of follicle cell populations, including terminal and stalk precursor cells.

**Conclusions:**

We demonstrate that with respect to mitotic cycle/endocycle switch Notch-signaling in *Tribolium* and *Drosophila* has opposing effects. While in *Drosophila* a Delta-signal brings about the follicle cells to leave mitosis, Notch-signaling in *Tribolium*is necessary to retain telotrophic egg-chambers in an “immature” state. In most instances, Notch-signaling is involved in maintaining undifferentiated (or preventing specialized) cell fates. Hence, the role of Notch in *Tribolium* may reflectthe ancestral function of Notch-signaling in insect oogenesis.

The functions of Notch-signaling in patterning the follicle cell epithelium suggest that *Tribolium* oogenesis may - analogous to *Drosophila* - involve the stepwise determination of different follicle cell populations. Moreover, our results imply that Notch-signaling may contribute at least to some aspects of oocyte polarization and AP axis also in telotrophic oogenesis.

## Introduction

The Notch family of receptors plays a central role in an evolutionarily conserved signaling pathway that regulates several cell fate decisions in many organisms [1]. *Notch* encodes a large transmembrane receptor for the ligands Delta (Dl), Serrate (Ser), which are also transmembrane proteins with large extracellular domains, and signaling therefore requires direct cell-cell contact. Insect oogenesis is one such example, where Notch-signaling regulates cell fate decisions of various cell types [1].

Establishment of distinct follicle cell fates at the early stages of *Drosophila* oogenesis is crucial for achieving the proper morphology of individual egg chambers [2,3]. Three distinct follicle cell populations are eventually defined: polar cells, which serve as key signaling centers, stalk cells, which will form the short bridge that connects neighboring egg chambers, and main-body follicle cells, which form an epithelium overlying the germline cyst. Polar and stalk cells are thought to arise from a common precursor population lineage [4-7]. Polar cell fate is induced in a restricted subset of this population by the Notch ligand Delta (Dl), which is produced in germline cells. Subsequently, Notch is required throughout the follicle cell epithelium to switch the main body cells from mitotic cell divisions to endoreplication [5,6].

Main-body follicle cells undergo three different modes of cell cycle [3]. From the germarium to stage 6, these cells undergo normal mitotic cycles. Beginning at around stage 7, main-body follicle cells undergo three rounds of endocycle (also called endoreplication), resulting in 16 copies of genomic DNA present in each nucleus. At stage 10B, genomic DNA replication stops, and the main body follicle cells switch from endoreplication to synchronized amplification of some genomic loci [8]. The amplified genomic regions encode eggshell proteins, which are required during late oogenesis. The transition from proliferation to endoreplication occurs when a Delta signal from the germline activates Notch in follicle cells. In egg chambers that contain either *Delta* germline clones or *Notch* follicle cell clones, follicle cells continue to proliferate beyond stage 6 [5,6].

Oogenesis in *Drosophila* and in the beetle *Tribolium* follows two different modes of oogenesis (out of three to be found among insects), the polytrophic-meroistic (*Drosophila*) and telotrophic-meroistic (*Tribolium*) oogenesis [9,10]. In polytrophic-meroistic ovaries each germ cell cluster matures as one unit encased by somatic follicle cells. While one cell of each cluster develops into an oocyte, the remaining 15 cells adopt nurse-cell fate. In telotrophic-meroistic ovaries as represented by the red flour beetle *Tribolium castaneum*, oocytes and nurse cells of a germ cell cluster separate such that each follicle contains only one growing germ cell, the oocyte. This oocyte remains connected to the tropharium – a syncytium of nurse cells – by a nutritive cord [10]. Arrested pro-oocytes are arranged around the somatic plug, a group of small somatic cells located at the posterior end of the tropharium. Pro-oocytes that lose contact to somatic plug cells enter the vitellarium and eventually become encapsulated by somatic follicle cells. During or shortly after encapsulation of the arrested pro-oocytes with somatic follicle cells, these so-called pre-vitellogenic egg chambers become arranged in a single row, depending on the activity of the JAK-STAT signaling pathway [9]. At that stage of *Tribolium* oogenesis an initial distinction of terminal/stalk precursor cells versus epithelial follicle takes place, as the linear arrangement of follicles probably requires differential adhesion characteristics for those follicle cells contacting two oocytes and those contacting only one. A subsequent step in *Tribolium* oogenesis then again involves JAK-STAT signaling and seems to result in the determination of stalk precursor cells out of a common terminal/stalk precursor population.

During vitellogenic growth of oocytes, interfollicular stalk cells become morphologically distinguishable. Oocytes considerably increase in size, while follicle cells divide until an average number of 1050 cells per follicle to form a uniform epithelial sheath surrounding the oocyte. Subsequently, follicle cell nuclei become polyploid and -after completion of vitellogenesis- secrete the eggshell, i.e. the vitelline membrane and the outer chorion [9,10].

To gain additional insides into the molecular mechanisms underlying telotrophic oogenesis, we have elucidated the functions of the Notch-signaling cascade in *Tribolium* oogenesis.

## Results and Discussion

### Expression of *tribolium notch* and *delta* in oogenesis

To monitor mRNA expression of *Tribolium Notch* and *Delta* during telotrophic oogenesis, we used the previously published clones [11]. *Tribolium Notch* is expressed ubiquitously in both germ line derived cells and somatic follicle cells (not shown). While Serrate is not expressed during *Tribolium* oogenesis, we found *Delta* expression to be restricted to the germ line (Additional file 1: Figure S1). In nurse cells and arrested pro-oocytes, *Delta* is expressed at low levels. At the time egg-chambers enter the vitellarium and during early pre-vitellogenic stages,*Delta* becomes strongly expressed in the oocytes. Subsequently, *Delta* levels decrease again. Expression of *Tribolium Delta* resembles the situation in *Drosophila*, where *Delta* signals from the germ line to induce distinct follicle cell fates, and later controls the transition from proliferation to endoreplication.

### *Depletion of notch* and *delta* affects egg-chamber formation and patterning

**Figure 1:**
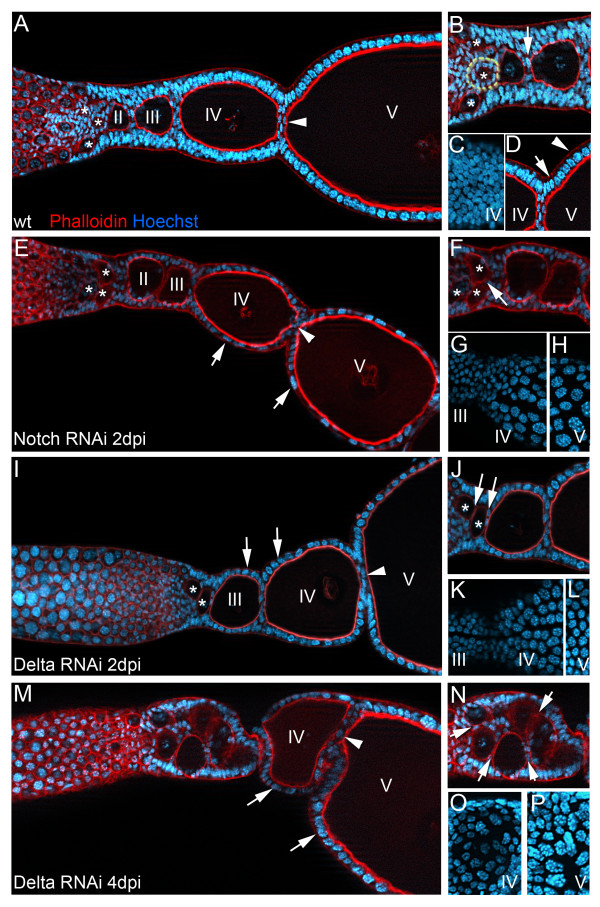
**Notch and Delta RNAi affects multiple steps in oogenesis.** Wildtype (A-D), *Notch* (E-H), and *Delta* RNAi two (I-L) and four days after dsRNA injection, respectively (M-P) (dpi). Roman numerals refer to egg-chambers in corresponding stages of oogenesis. Ovarioles were stained for f-actin with Phalloidin (red) andfor DNA with Hoechst. (blue). In panels C,G,H,K,L,O,P only the Hoechst staining is shown. Panels (B-D), (F-H), J-L), and (N-P) are close ups of the ovarioles shown in A,E,I, and M, respectively. (**A-D**) In wildtype, pro-oocytes (A, asterisks) enter the vitellarium and eventually become encapsulated by somatic follicle cells (B, dotted line). Individual follicles become subsequently separated by interfollicular stalk cells (A, arrowhead; B, arrow). As judged by morphology (i.e. size of the nuclei) (C, top view of the follicle cell epithelium of the ovariole in A), follicle cell nuclei are still in mitotic cycle. During late stages of oogenesis, yolk is taken up by the oocyte from the hemolymph (vitellogenesis). The vitellogenic oocyte considerably increases in size, while follicle cell nuclei switched from mitotic (D, arrow) to endocycle (D, arrowhead). (**E-H**) Two days after injection of *Notch* dsRNA, the follicle cell epithelium appears disorganized (E, arrows). Interfollicular stalks are absent (E, arrowhead) and compound follicles (i.e. egg-chambers that comprise of more than two oocytes) are observed (F, arrow). Already at pre-vitellogenic stages, follicle cell nuclei are increased in size (E, arrows; G,H, top views of the follicle cell epithelium of the ovariole in E), indicating that the nuclei have become polyploid (compare C to G). (**I-L**) Also upon *Delta* RNAi, follicle cell nuclei seem to become polyploid prematurely (I, arrows; K,L top view of the follicle cell epithelium of the ovariole in I). The follicle cell epithelium appears rather patchy (I, arrows), stalk cell were missing (I, arrowhead), and compound follicles are observed (J, arrows). (**M-P**) Four days after injection, *Delta* RNAi phenotypes are increased in strength. Oocytes accumulate in large compound follicle (I,N) and also the size of follicle cell nuclei further increases (M, arrows; O,P, top views of the follicle cell epithelium of the ovariole in M). Egg-chambers seem to maximize their area of contact (M, arrowhead). All panels: anterior to the left. Asterisks label pro-oocytes that enter the vitellarium.

During *Drosophila* oogenesis, Notch signaling is important for the formation of several follicle cell lineages [4-7,12]. Subsequently, Notch is required throughout the follicle cell epithelium to switch from mitotic cell divisions to endocycle. To elucidate, whether telotrophic oogenesis also requires the activity of the Notch-pathway, we knocked down *Tribolium Notch* and *Delta* by adult RNAi and dissected ovaries at different time points after injection (days post injection, dpi). Adult RNAi against *Tribolium Notch* and *Delta* results in severe but basically identical phenotypes (Figure 1), which were not further enhanced by the combined knockdown of both genes (not shown). Two to four days after dsRNA injection, the follicle cell epithelium appeared patchy and disorganized as compared to wild type (Figure 1 E,I,M), which was accompanied by a block of oogenesis, as we observed the cessation of egg production in these females. As compared to wildtype, follicle cell number appeared to be deceased in *Notch* or *Delta* RNAi and moreover, follicle cell nuclei were increased in size (Figure 1G,H,K,O), indicating that follicle cell nuclei of pre-vitellogenic and early vitellogenic egg-chambers became polyploid, i.e. switched to endoreplication (see below).

### Notch-signaling is required to maintain the mitotic cycle

**Figure 2:**
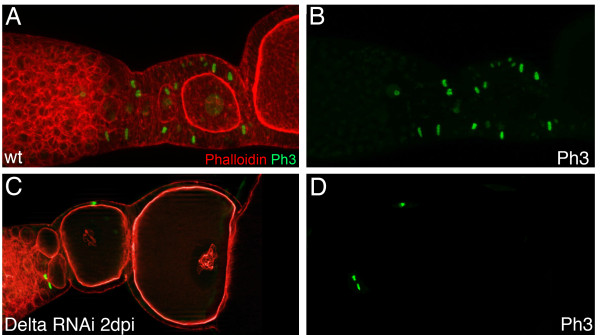
**In *Delta w*RNAi the rate of follicle cell mitosis is decreased. **Wildtype (A,B) and*Delta *RNAi (C,D) two days after dsRNA injection stained for f-actin with Phalloidin (red) and for an antibody recognizing phosphorylated Histone-3 (green). (B,D Anti-phospho-histone (PH3) channel only). (**A,B**) In wildtype, cell divisions are restricted to early follicle cells and rather scattered than concentrated to distinct regions. Mitosis can be found in the posterior part of the tropharium during early follicle growth and also in those follicle cells that may contribute to stalk formation. (**C,D**) Upon *Delta *RNAi, follicle cell cease mitosis.

To uncover the effects of Notch-signaling on follicle cell patterning, mitosis and the switch to endocycle, we observed morphological and genetic markers. First, we monitored cell division patterns by a cross-reacting antibody against phosphorylated histone-3 (pH3). In wildtype follicles, an average number of 30 mitotically active cells per ovariole was observed (Table 1, Figure 2). During telotrophic *Tribolium* oogenesis, cell proliferation is restricted to somatic cells and most dividing cells are scattered among the follicle cell epithelium without being limited to obvious domains [9]. Upon *Delta* RNAi, most follicle cells cease mitosis and the number of cell divisions drops to an average number of 14 per ovariole.

**Table 1:** Number of mitotic cell

**Ovariole**	**WT**	***Delta-*RNAi**
1	31	8
2	39	8
3	25	23
4	27	12
5	37	19
6	20	14
7	30	17
8	27	19
9	32	19
10	30	5
11	27	11
Average:	**30**	**14**

The number of mitotic follicle cells in wildtypeand*Delta* RNAi ovarioles was determined by a cross-reacting antibody against phosphorylated histone-3 (pH3). For every experiment, eleven ovarioles were scored for Ph3 signals.

**Figure 3:**
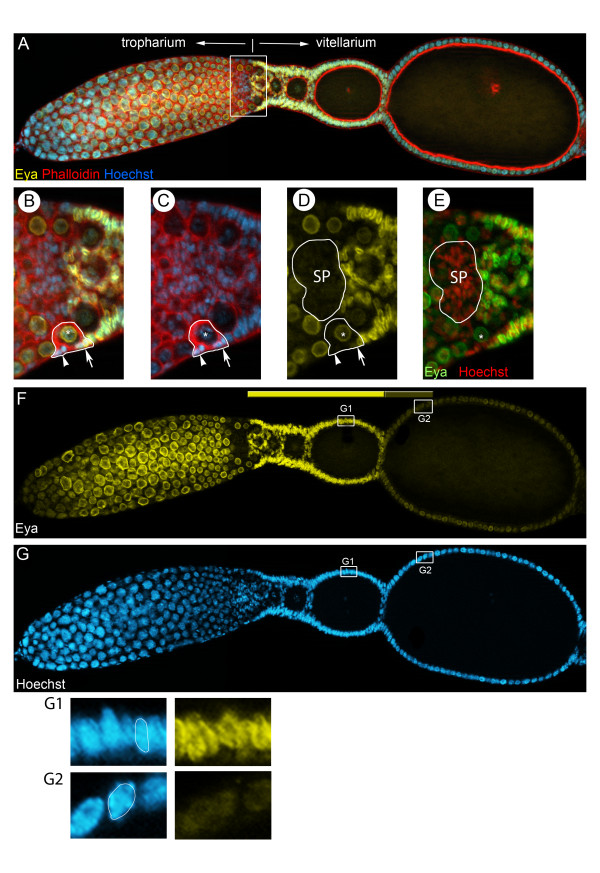
**Eyes-absent as marker for the switch from regular mitosis to endocycle.** (A-G) Wildtype ovariole stained for f-actin with Phalloidin (red), DNA with Hoechst (blue), and for a cross reacting antibody against Eyes-absent (Eya) (yellow). Panels B-E are close ups of the ovariole shown in A. (E) The Eya pattern is shown in green. In (F) only the Eya channel and in (G) only the Hoechst channel is shown. Panels (G1-G2) are close ups of the ovariole shown in (F,G); nuclei stained for Hoechst and Eya. (**A**) Eya is expressed in germline derived cells of the tropharium and in a subset of somatic cells. (**B-E**) In the vitellarium, Eya expression first can be observed upon pro-oocyte encapsulation. Pre-follicular cells, which successively encapsulate the oocytes to form an egg-chamber, start expressing Eya at high levels (B-D; arrowhead, arrow). Note that pro-oocytes are also Eya positive (asterisk in B-D). Subsequently, all follicle cells express Eya. Interestingly, Eya is absent from somatic plug cells (SP) (D,E). (**F-G**) During mitotic stages, Eya is continuously expressed at high levels (F, G1). At the time follicle cell cease mitosis and enter the endocycle, follicle cell nuclei increase in size and are no longer columnar, but rather round (compare G1 to G2). At that time Eya signals get significantly weaker(F, compare G1 to G2). Hence, only mitotically active follicle cells express Eya at high levels.

**Figure 4:**
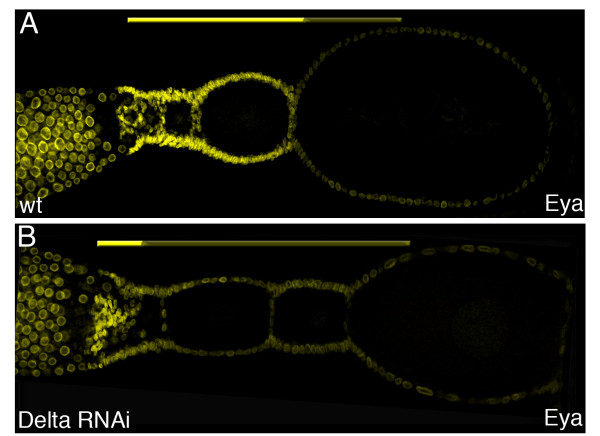
**Eya expression in *Delta *RNAi. **(A-C) Eya expression in (**A**) Wildtype and (**B**) *Delta *RNAi. (**A**) In Wildtype ovarioles, Eya expression is restricted to mitotically active follicle cells (see fig. 4 for details). (**B**) In Delta depleted ovarioles, Eya expression is severely reduced. Only in the anterior region of the vitellarium, a few follicle cells express Eya at high levels.

Our results indicate that *Delta* depleted follicle cells cease mitosis too early. To elucidate whether the reduction in mitotic activity in *Delta* RNAi is related to a premature switch from mitotic to endocycle, we monitored the expression of Eyes-absent (Eya) by a cross-reacting antibody (Figures 3 and 4). Eya is a nuclear protein best known for its role in *Drosophila* eye development. During polytrophic *Drosophila* oogenesis, Eya serves as repressor of polar and stalk development and Eya has to bedown regulated for these fates to be determined [13]. In *Tribolium*, Eya expression can be observed in the nuclei of germ-line derived cells in the tropharium (Figure 3A,F). In the vitellarium, however, Eya is restricted to follicle cells (Figure 3B-F). Interestingly, Eya is expressed at high levels in mitotically active follicle cells, while at the time follicle cell nuclei become polyploid, Eya expression ceases, indicating that Eya is down regulated at the time follicle cells switch from regular mitosis to endocycle (Figure 3G). Hence, in *Tribolium* Eya expression serves a suitable marker for the transition of follicle cells from proliferation to endoreplication.

In *Delta* RNAi ovarioles, Eya expression in follicle cells is severely reduced (Figure 4B). Only a few follicle cells in the transition zone between tropharium and vitellarium still express Eya at high levels. The far majority of follicle cells, however, only display low level of Eya, supporting our assumption that in absence of Notch-signaling follicle cells enter endocycles prematurely, which obviously prevents additional cell divisions. In contrast to *Drosophila*, were a Notch-signal brings about the follicle cells to leave mitosis [5,6], in *Tribolium* egg-chambers, Notch-signaling apparently prevents follicle cell from entering endocycle. Thus with respect to the cycle/endocycle switch, Notch-signaling in *Tribolium* and *Drosophila* has opposing effects.

### *Delta* RNAi prevents proper oocyte encapsulation

The first important function of Notch-signaling in *Drosophila* oogenesis is encapsulation of the cyst by follicle cells, which is required to establish distinct follicle cell fates at the early stages of oogenesis. In *Notch* mutant flies, adjacent egg chambers are often fused giving rise to compound egg chambers that contain multiple germ line cysts in a single follicular epithelium [14,15]. Also in *Tribolium*, the depletion of *Notch* and *Delta* results in the formation of compound follicles and affects shape and size of egg-chambers (Figure 1). Therefore we elucidated, whether the impact of *Delta* RNAi on oocyte encapsulation and follicle architecture is due to the premature termination of proliferation in *Delta* RNAi, or if Notch-signaling is indeed involved in the specification of different follicle cell populations.

**Figure 5:**
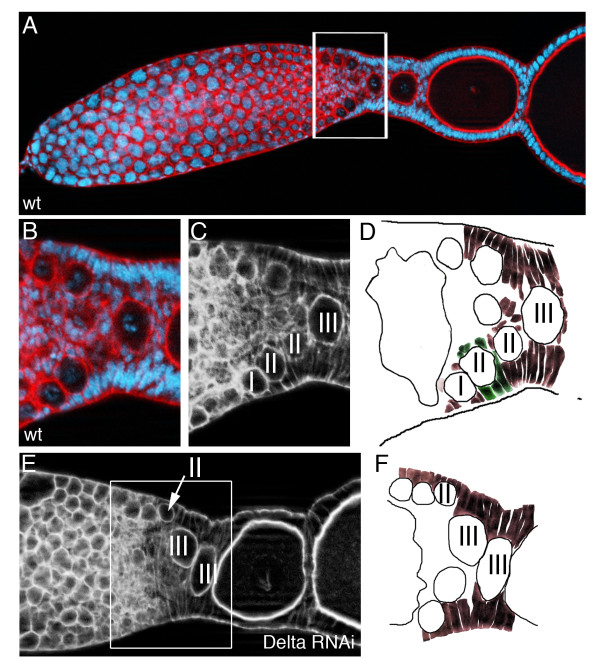
***Delta *RNAi affects encapsulation. **Wildtype (**A**) and *Delta *(**E**) RNAi ovarioles stained for f-actin with Phalloidin (red), DNA with Hoechst (blue). (**C,E**) phalloidin channel only. Roman numerals refer to egg-chambers in corresponding stages of oogenesis. (**B-D**) Close up of the wildtype ovariole shown in (A) (**D**, schematic representation). During encapsulation, pro-oocytes (I) first come in contact with lateral pre-follicular cells, which successively engulf the oocytes to form an egg-chamber (II). As these early follicles develop, they become aligned in a single row (III). (**E,F**) In *Delta *RNAi, encapsulation is incomplete (compare stage III egg-chambers in D to F). Pro-oocytes are not separated from each other, resulting in egg chambers comprising of multiple oocytes.

In the wild type *Tribolium* ovary, arrested pro-oocytes are arranged around the somatic plug (Figure 5) [9]. Upon pro-oocyte maturation, these cells separate from the somatic plug and enter the vitellarium, where they become encapsulated by somatic cells (Figure 5B-D). During this process, pro-oocytes first come in contact with lateral pre-follicular cells, which successively encapsulate the oocyte to form an egg-chamber (Figure 5D). As these early follicles develop, they become aligned in a single row. In *Delta* RNAi, encapsulation is incomplete (Figure 5E,F). As indicated in Figure 5, maturating pro-oocytes are in contact with pre-follicular cells. Subsequent stages of encapsulation, however, were not observed, ultimately resulting in egg chambers comprising of multiple oocytes (Figure 1M and Figure 5E). As judged by morphology, in *Delta* RNAi the number of pre-follicular cells is reduced. Hence, while follicle cells enter the endocycle premature following *Delta* RNAi, there might be not enough follicle cells for proper egg-chamber formation.

### Notch-signaling in follicle cell patterning

**Figure 6:**
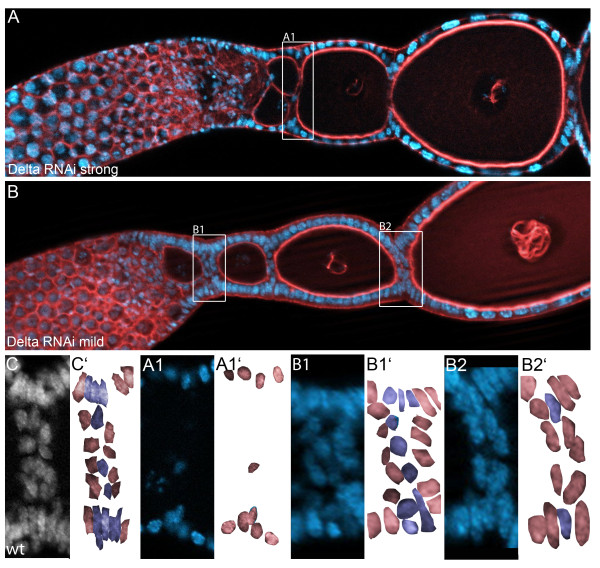
**In *Delta *RNAi terminal follicle cell populations are reduced. **Strongly (A) and mildly (B) affected Delta RNAi ovarioles stained for f-actin with Phalloidin (red), DNA with Hoechst (blue). (C)Wildtype stalk stained for DNA with Hoechst (C’ schematic representation). Stalk cells (blue cells in C’) separate individual egg-chambers (red cells in C’) and do not contact the germ line. (**A, A1**) In severely affected *Delta *RNAi ovarioles, stalk and terminal follicle cell are absent (A1 close up of the ovariole in A; A1’ schematic representation), while lateral follicle cells are basically present. (**B, B1, B2**) In weak *Delta *RNAi phenotypes, the follicle cell epithelium appears rather normal (B1 and B2 close ups of the ovariole in B; B1’ and B2’schematic representations). Still, stalk cells are reduced (B2) in number, indicating a function for Notch-signaling in the determination of stalk cells.

During vitellogenic growth of the oocyte interfollicular stalk cells, which separate individual egg-chambers, become morphologically distinguishable. Initially, only a few cells separate the egg chambers. Successively, differentiated stalk cells can be recognized by their position (i.e. stalk cells do not contact the germ line) and by their characteristic cellular and nuclear morphology (i.e. disc-shaped). As judged by morphology, in mildly affected *Delta* RNAi ovarioles, the number of stalk cells is severely reduced (Figure 6B). Still, individual egg-chambers remained separated by two layers of follicle cells, and thus were encapsulated properly. As described before, in strongly affected *Delta* RNAi ovarioles the number of follicle cells is reduced and the follicle cell epithelium appears disorganized, resulting in egg chambers comprising of two or more oocytes (compound follicles). Interestingly, however, while we still observed residual lateral follicle cells, terminal follicle and stalk cells were largely absent (Figure 6A), indicating that mild *Delta* RNAi phenotypes are not solely due to the premature termination of cell proliferation. Therefore, we asked whether Notch-signaling is indeed involved in the specification of terminal and stalk follicle cell populations.

**Figure 7:**
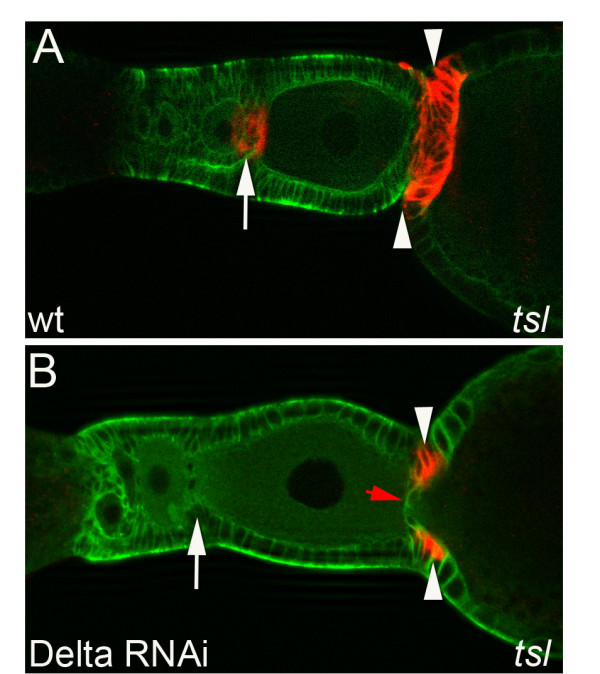
***torso-like *expression in *Delta *RNAi.**Wildtype (A) and *Delta *(B) RNAi ovarioles stained for tubulin (green) and *tsl* mRNA (red). (**A**) In wildtype *tsl *is expressed in the interfollicular stalk (arrowheads) and in putative stalk precursor cells (arrow). (**B**) Upon *Delta *RNAi, *tsl *mRNA in stalk cells is severely reduced (arrowheads), while it is absent in the stalk precursors (arrow). Still some residual differentiated stalk cells remain (red arrow).

Previously, we have shown that stalk cell lineage can be monitored by *torso-like* (*tsl)* expression [9]. Initially, *tsl* is expressed by the putative stalk precursor population and subsequently (Figure 7A, arrow), *tsl* expression can be observed in differentiated stalk cells, as well as in adjacent cells, which contribute to the separation of individual follicles (Figure 7A, arrowhead). We monitored *tsl* expression in mildly affected *Delta* RNAi ovarioles (i.e. two days after injection of Delta dsRNA). As judged by morphology, there are still differentiated stalk cells (Figure 7 B, red arrow), as well as stalk precursor cells (Figure 7B, white arrow) present.

While the remaining differentiated stalk cells lost *tsl* expression (Figure 7 B, red arrow), some of the adjacent cells (i.e. non-stalk cells) were still *tsl* positive (Figure 7B, arrowheads). Strikingly, we found *tsl* expression also to be lost from putative stalk precursor cells (Figure 7B, white arrow), indicating a function for Notch-signaling in specifying the stalk cell lineage.

In *Drosophila*, follicle cell lineages are established in stepwise manner. Polar cell fate is induced in a restricted subset of this population by Delta, which is produced in germline cells [5,6]. Polar cells, in turn, express the ligand Unpaired (Upd; Outstretched), which activates the JAK/STAT signaling pathway in neighboring polar/stalk precursors, thereby inducing the stalk cell fate [4,16-18]. Also in telotrophic *Tribolium* oogenesis, stalk formation involves the determination of stalk precursor cells out of a common terminal/stalk cell population in a JAK-STAT dependent manner. In *dome* or *STAT* RNAi, stalks cells are lost and individual egg-chambers maximize the area of contact [9]. In *Delta* RNAi, however, not only stalk and stalk precursor cells, but the entire terminal follicle population is lost, indicating that - analogous to *Drosophila*-Notch-signaling in telotrophic oogenesis is required for early steps in follicle cell patterning, i.e. the determination of terminal follicle cells. Subsequently, graded levels of JAK-STAT signaling may specify additional follicle cell populations, including stalk precursor cells. Therefore we posit that early steps in follicle cell patterning are conserved between polytrophic and telotrophic oogenesis, as both involved the specification of follicle cell populations in stepwise and JAK-STAT and Notch dependent manner.

### Axis formation in telotrophic oogenesis

**Figure 8:**
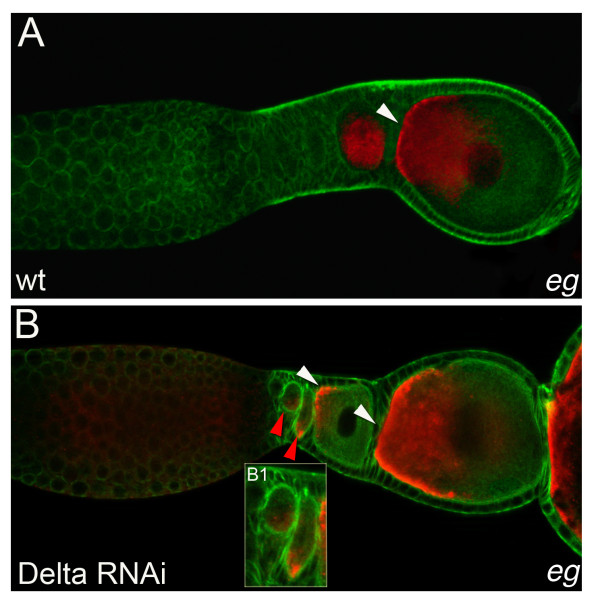
***eagle *mRNA localisation in *Delta *RNAi ovarioles.** Wildtype (A) and *Delta *RNAi (B) ovarioles stained for tubulin (green) and *eagle *mRNA (red). (**A**) In Wildtype ovarioles, *eg *mRNA becomes restricted to the anterior part of the oocyte (arrowheads). (**B**) Four days after dsRNA injection the *Delta *phenotype is obvious, as the stalk is absent and egg chambers are directly attached to each other. While, in older egg-chambers transcripts become localized to the anterior pole of the oocyte (white arrowheads), in early egg-chambers mRNA localization occurs premature and perpendicular to the apparent anterior-posterior axis of the egg-chamber (red arrowheads, B1 close up of the ovariole in B).

Next we exaimined, whether Notch-signaling is required for anterior-posterior axis formation during *Tribolium* oogenesis. To this end, we monitored the expression of *eagle* (*eg*) mRNA in *Delta* depleted ovarioles (Figure 8). In wildtype ovarioles, *eg* mRNA initially is expressed ubiquitously and subsequently, becomes localized to the anterior pole of vitellogenic oocytes (Figure 8), indicating a polarization event at that stage of oogenesis [9].

Interestingly, also in *Delta *depleted ovarioles we still observed the localization of *eg*mRNA. However, while in older egg-chambers transcripts become localized to the anterior pole of the oocyte, in young follicles mRNA localization frequently occurs premature and perpendicular to the apparent anterior-posterior axis of the egg-chamber (Figure 8B).

In *Drosophila *,axes formation depends on a series of cell-cell-signaling events between the germline and the soma and between different populations of somatic cells (for review see [19,20]). The first symmetry-breaking step in *Drosophila* oogenesis is a Delta signal given out by the germline cyst as it buds from the germarium [4,6]. This induces the adjacent anterior follicle cells to differentiate into polar cells, which express the JAK/STAT ligand Upd and eventually induces terminal follicle cell fates, including stalk cell [18,21]. Stalk formation induces the follicle cells anterior to it, which contact the neighboring younger germline cyst, to upregulate E-cadherin. This allows them to adhere preferentially to the posterior pole of the oocyte in the younger cyst, thereby positioning it posterior to the nurse cells [22,23].

In response to a second Delta signal from the germline, cells become competent to respond to subsequent inductive signals. The anterior and posterior polar follicle cells continue to secrete Unpaired and this gradient distinguishes terminal follicle cells from main-body follicle cells and establishes a symmetrical pre-pattern of the follicular epithelium [18,21]. This (AP) symmetry is broken when the oocyte nucleus lies close to the posterior pole of the oocyte, where EGF signaling to the overlying posterior follicle cells, and subsequent back signaling, lead to the repolarisation of the oocyte cytoskeleton and to the localization of maternal *bicoid *and *oskar *mRNAs at the poles [24].

The premature polarization of the oocyte in absence of *Delta* indicates that also in *Tribolium *interactions of the germline and the soma contribute at least to the timing of oocyte polarization. Since *Delta *depleted follicle cells enter endocycles prematurely, it is imaginable that the switch from mitosis to endoreplication triggers oocyte polarization and eventually, mRNA localization. However, in wildtype ovarioles there is no correlation of the onset of *eg *mRNA localization and the switch to endocycle (mRNA localization precedes endoreplication; not shown). Still, our results suggest that in contrast to *Drosophila* oogenesis, Notch-signaling is required to keep telotrophic egg-chambers in an “immature” or “unpolarized” state.

In addition to the premature polarization of the oocyte we also observed the miss-localization of *eg* mRNA with respect to the anterior-posterior axis of the egg-chamber, suggesting that Notch-signaling may indeed influence the determination of the anterior posterior axis. In *Drosophila*, the positioning of the oocyte at the posterior of the germline cyst ultimately leads to the formation of the A-P axis of the embryo [20]. The oocyte is positioned by a Notch-dependent relay mechanism that includes a series of posterior to anterior inductions. The older cyst induces the anterior polar cells, the anterior polar cells induce the stalk, and the stalk induces the positioning of the oocyte of the younger anterior cyst [7]. Given that terminal follicle cell populations (including stalk cells) are lost in *Tribolium* in *Delta* RNAi, interaction of posterior and anterior follicle might be interrupted, and as a consequence, oocytes are not polarized correctly. Thus, it is tempting to speculate that also telotrophic oogenesis involves a Notch-based relay mechanism signaling from older to younger follicles, similar to the way follicle polarity is transmitted in *Drosophila* from one cyst to the next younger one [7].

Still, we cannot exclude that the miss-localization of *eg* transcripts rather is due to the displacement or rotation of egg-chambers. Previously, we have shown that the depletion of JAK-STAT also affects the alignment of pre-vitellogenic egg chambers. In wildtype ovarioles, early follicles become arranged in a single row as they are engulfed by somatic cells. Upon JAK-STAT RNAi, young oocytes still are encapsulated by follicle cells, but are not aligned in a linear row, which eventually results in the displacement of entire-egg chambers. Even though in *Delta* RNAi egg-chambers still become aligned linearly, encapsulation is incomplete, which may also result in the rotation of follicles. Thus, miss-localization of *eg* transcript could be due to effects of *Delta* RNAi in follicle alignment and orientation within the ovariole.

The polarization of the oocyte in telotrophic *Tribolium* oogenesis is - in contrast *Drosophila* - apparently independent of JAK-STAT signaling and also the *Tribolium* Gurken (*grk*) orthologue seems not to play a major role in AP axis formation, but rather acts in dorso-ventral patterning [25]. Even though our results suggest that Notch-signaling might contribute at least to some aspects of oocyte polarization also in *Tribolium*, the mechanisms of AP axis formation in telotrophic oogenesis remain to be elucidated.

## Conclusions

Notch-signaling is used widely to restrict particular fate choices and to regulate pattern formation throughout the animal kingdom. In this study we show that Notch-signaling has at least two distinct functions during telotrophic *Tribolium* oogenesis, Notch-signaling is required to keep follicle cells in the mitotic cell cycle. Consequently, the loss of Delta or Notch triggers premature endoreplication of follicle cells. While with respect to mitotic cycle/endocycle switch Notch-signaling in *Tribolium* and *Drosophila* has opposing effects, the function of Notch-signaling in *Tribolium* may reflect the ancestral role of Notch-signaling in restricting cell fates.

In addition, we provide evidence that Notch-signaling is involved in the determination of distinct follicle cell fates also in the *Tribolium* ovary. As judged by morphology and *tsl* expression, in mildly affected *Delta* RNAi ovarioles terminal but not lateral follicle cell populations are severely affected, indicating that those phenotypes are not solely due to the premature termination of cell proliferation. We posit that function of Notch-signaling in follicle cell patterning during telotrophic oogenesis resembles the situation in *Drosophila*, and thus, may represent an ancestral feature of epithelial pattern formation in insect oogenesis.

We found conserved and divergent aspects of follicle cell patterning in telotrophic *Tribolium* oogenesis. Given that in most instances, Notch-signaling is involved in maintaining undifferentiated (or preventing specialized)cell fates, the role of Notch in *Tribolium* may reflect the ancestral function of Notch-signaling in insect oogenesis.

## Methods

For *Tribolium Notch*, *Delta,* and *Serrate*, we used the previously published clones [11]. Dissection and fixation of female gonads were performed as described before [9,10].

To visualize the morphology, Hoechst 33258 and TRITC labelled Phalloidin, which labels the f-actin cytoskeleton were used. Whole mount in situ hybridization, were essentially performed as described before [9].

To visualize the morphology after in situ hybridization, ovaries were counterstained by Hoechst 33258 (5 μg/ml) and an anti-α-Tubulin antibody (mouse monoclonal, 1:1000; Sigma). Mitotically active cells were labelled by an anti-phospho-Histone H3 antibody (rabbit polyclonal, 1:200; Upstate). The mouse monoclonal anti-Eyes-absent antibody (DSHB,) was used in dilution of 1:100. The following secondary antibodies were used: Cy2 or Cy3 conjugated goat anti-rabbit (Jackson Immuno-Research, 1:50); Cy2 or Cy3-conjugated sheep anti-mouse (1:200, Sigma). All incubations were done at 4°C over night. Ovary images were captured on a Zeiss ApoTome fluorescence microscope.

## Competing interests

The authors declare that they have no competing interests.

## Authors' contributions

NS and DB carried out the study. MS conceived the study and wrote the manuscript. All authors read and approved the final manuscript.

## Supplementary Material

Additional file 1 **Figure S1.** Expression of *Tribolium* Delta. Click here for file
