# Supplementary material for: Opposing effects of Notch-signaling in maintaining the proliferative state of follicle cells in the telotrophic ovary of the beetle Tribolium
Source: Front Zool. 2012 Aug 6;9:15. doi: 10.1186/1742-9994-9-15 (PMC3502128; doi:10.1186/1742-9994-9-15)

**Supplementary Figure**

**Figure S1: Expression of *Tribolium* Delta**

**(A)** Wildtype ovariole stained for *Delta* mRNA. *Delta* expression is restricted to the germ line. In nurse cells and arrested pro-oocytes, *Delta* is expressed at low levels. At the time egg-chambers enter the vitellarium and during early pre-vitellogenic stages, *Delta* becomes strongly expressed in the oocytes (arrowheads). Subsequently, *Delta* levels decrease again (arrow).


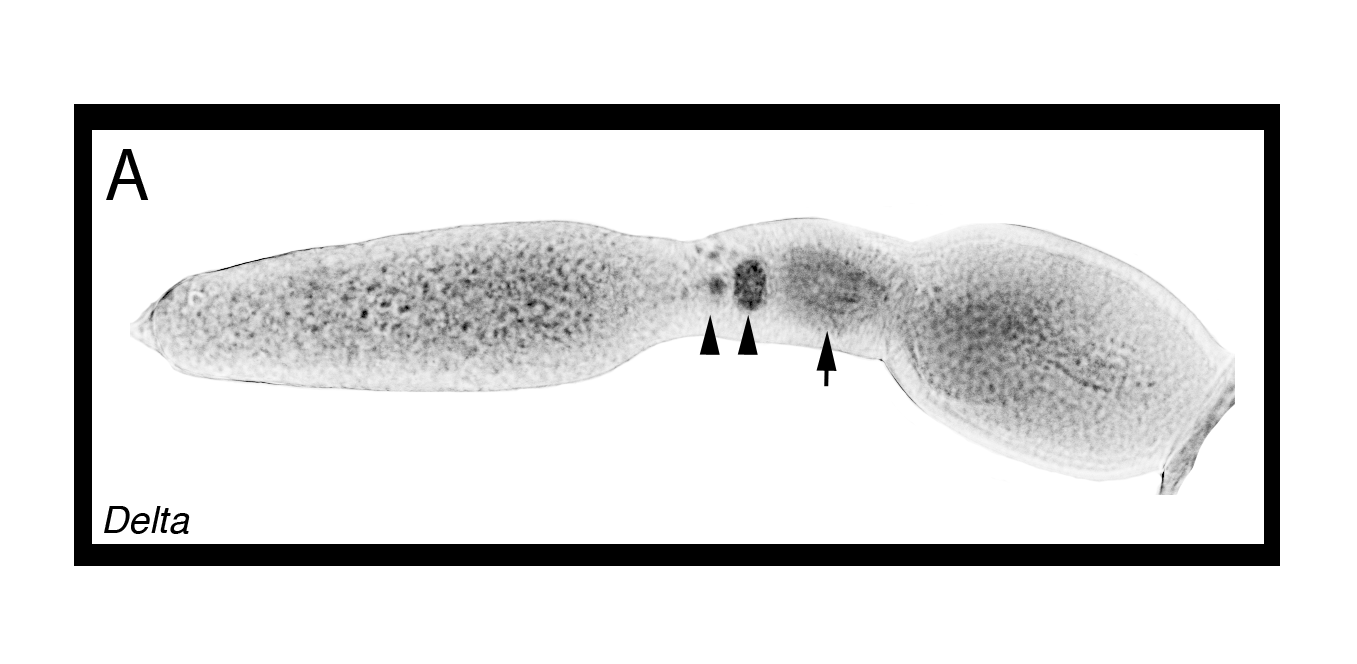

Supplement: Additional file 1 — Figure S1. Expression of Tribolium Delta. [file 1742-9994-9-15-S1.docx]
